# Endoscopic-Ultrasound-Guided Gallbladder Drainage in Patients with Percutaneous Cholecystostomy Drain

**DOI:** 10.3390/jcm15114367

**Published:** 2026-06-05

**Authors:** Raahi Patel, Mohamed Ebrahim, Varshita Goduguchinta, Ahamed Khalyfa, Khalil Ur Rehman, Navkiran Randhawa, Mahnoor Inamullah, Rahil Desai, Kamran Ayub

**Affiliations:** 1 Department of Internal Medicine, Franciscan Health, Olympia Fields, IL 60461, USA; raahip07@gmail.com (R.P.); vgoduguchinta1@gmail.com (V.G.); 2 Department of Internal Medicine, Ascension Saint Joseph Hospital, Chicago, IL 60657, USA; mohdaymanebrahim@gmail.com; 3 Department of Gastroenterology, The University of Iowa, Iowa City, IA 52242, USA; akhalyfa1@gmail.com; 4 Department of Internal Medicine, Carle Foundation Hospital, Urbana, IL 61801, USA; khalilurrehmantajik@gmail.com; 5 Department of Gastroenterology, Augusta University, Augusta, GA 30912, USA; randhnk@gmail.com; 6 Southwest Gastroenterology, a Division of GI Partners of Illinois, Oak Lawn, IL 60453, USA; mahnoor.fm93@gmail.com; 7 Department of Gastroenterology, Advocate Lutheran General Hospital, Park Ridge, IL 60068, USA; rahildesai@gmail.com; 8 Department of Gastroenterology, Silver Cross Hospital, 1900 Silver Cross Blvd, New Lenox, IL 60451, USA

**Keywords:** percutaneous cholecystostomy, endoscopic ultrasound-guided gallbladder drainage, laparoscopic cholecystectomy, lumen-apposing metal stent

## Abstract

**Background/Objectives**: Laparoscopic cholecystectomy (LC) is the current gold standard in patients with acute cholecystitis. Percutaneous cholecystostomy (PC) remains an option for those who are not surgical candidates but is associated with adverse effects. We studied technical success and patient satisfaction for endoscopic ultrasound-guided gallbladder drainage (EUS-GBD) after initially receiving a PC drain. **Methods**: A multi-center study was conducted at 4 institutions involving patients who initially received a PC. These patients were given the option to transition to receive EUS-GBD. A 5-point Likert scale was used to assess patient satisfaction comparing PC vs. EUS-GBD. Demographic data, including age, sex, reason for PC, complications, and patient satisfaction scores, were collected. **Result**: All seven patients who underwent percutaneous cholecystostomy rated their experience as 1 (very dissatisfied), whereas the same patients rated EUS-guided gallbladder drainage with a mean satisfaction score of 4.7 (very satisfied). **Conclusions**: EUS-GBD is effective and offers higher satisfaction scores in patients who are not surgical candidates.

## 1. Introduction

Acute cholecystitis (AC) is defined as inflammation of the gallbladder precipitated by a blockage of the cystic duct. Around 200,000 people annually are diagnosed with acute cholecystitis in the United States of America [1]. The typical presentation consists of right upper quadrant abdominal pain that can radiate to the back or shoulder, bloating, nausea, vomiting and fever. Diagnostic modalities include obtaining a right upper quadrant ultrasound, which typically shows a distended gallbladder wall, pericholecystic fluid, and a sonographic Murphy’s sign [2]. However, if ultrasound findings are not conclusive, a hepatobiliary iminodiacetic acid scan (HIDA scan) could be used. This is conducted by administering an intravenous injection of radiolabeled hydroxy iminodiacetic acid, followed by scanning the abdomen. The tracer is followed through the gallbladder and bile duct and analyzed to see if there are any blockages [2].

Once a diagnosis has been established, the treatment regimen is most commonly surgical intervention. The first-line therapy is performing a laparoscopic cholecystectomy (LC), typically within 3 days of an established diagnosis, if there are no complications such as cholangitis [1]. Wu et al. conducted a meta-analysis comparing early versus delayed LC for AC in 1625 patients. They found that early LC was associated with lower hospital costs, fewer workdays lost, higher patient satisfaction and quality of life, lower risk of wound infection and shorter hospital stay [3]. However, in some cases, surgical intervention could be contraindicated due to other comorbidities.

There are multiple scoring tools available to assess the severity of a patient’s AC and the patient’s ability to withstand surgery. The Tokyo guidelines were specifically created to provide diagnostic criteria and severity grading for AC [4]. This scoring criterion looks at local and systemic signs of inflammation, imaging findings, and other signs of organ dysfunction to determine the score [5]. The severity grading criteria are broken down as mild AC (Grade I), moderate AC (Grade II), and severe AC (Grade III). While grade I severity allows for cholecystectomy to be performed as a safe and low-risk procedure, grade II severity is associated with increased operative difficulty during cholecystectomy [6]. Grade III severity is associated with organ dysfunction. Another tool called the American Society of Anesthesiologists (ASA) score helps assess a patient’s health prior to receiving anesthesia and undergoing surgery. The score is measured between 1 and 6, with 1 being a healthy patient and 6 being a brain-dead patient [7]. A study conducted by Osterman et al. studied 725 patients who underwent emergency LC. Complications occurred in 9% of ASA1, 13% of ASA2, and 24% of ASA3 patients, highlighting the correlation between a higher ASA score and an increased risk of undergoing surgery [7].

The aforementioned scores allow clinicians to make decisions on whether a patient is a surgical candidate or to pursue non-operative management (NOM). The use of antibiotics and analgesics is part of NOM. Some of the non-surgical options for patients presenting with AC include percutaneous cholecystostomy (PC), percutaneous transhepatic gallbladder drainage (PTGBD), and endoscopic ultrasound-guided gallbladder drainage (EUS-GBD). PC, which is a drain placed in the gallbladder, is the current recommended method of choice in non-surgical candidates but is associated with higher rates of complications such as bile leak and biliary peritonitis [8]. In addition to drain placement, a delayed LC is still recommended once a patient’s clinical condition improves. EUS-GBD is another option for gallbladder drainage that is relatively novel and a good alternative to PC, especially in high-risk surgical candidates. It involves endoscopic evaluation with internal drainage of the gallbladder, typically with the use of a lumen-apposing metal stent placement. While there are some studies favoring the use of EUS-GBD over PC in regard to overall outcomes, there is limited data providing well-defined protocols. There is also very limited data regarding patient satisfaction.

We present a case series involving seven patients who initially had a PC placed but were given the option to convert to an EUS-guided drainage of the gallbladder and removal of the drain due to ongoing symptoms or recurrent infections of the drain.

## 2. Materials and Methods

This retrospective case series, conducted across four tertiary care institutions (3 Silver Cross Hospital, 2 Advocate Christ Hospital, 1 Advocate Good Samaritan Hospital, 1 St. Joe Medical Center) specializing in advanced endoscopic and hepatobiliary interventions, evaluated seven patients, with a mean age of 74 years (range 48–92); 2 males, 5 females who had initially undergone PC for AC but were subsequently offered conversion to EUS-GBD due to dissatisfaction with external drainage (Table 1). All 7 patients received antibiotics for AC. Specifically, 4 patients received amoxicillin/clavulanate, 2 received ciprofloxacin and 1 received piperacillin-tazobactam. Exactly 7 patients were screened and offered EUS-GBD consecutively and evaluated in an outpatient setting. The shortest time from PC to EUS-GBD was 4 weeks.

The patient cohort represented a challenging population with multiple comorbidities, including five patients with advanced malignancies (three patients with pancreatic cancer metastases, one with hepatocellular carcinoma, and one with breast cancer metastases), decompensated cirrhosis (one patient), and significant cardiopulmonary disease (one patient with COPD, CAD, and CHF). The elderly nature of the population reflects their high-risk profiles, as they were not deemed surgical candidates at the time of evaluation for PC. Specifically, 3 patients were deemed ASA3, while 4 patients were deemed ASA4. This was determined by the tertiary center surgeons who had initially evaluated the patients. The primary indications for converting to EUS-guided gallbladder drainage included recurrent complications from percutaneous cholecystostomy drains and pain due to the drain. Among the seven patients, five had pain-related indications: pain with excoriation at the drain site (n = 1), pain with drain malfunction (n = 1), pain with site infection (n = 1), pain leading to patient intolerance (n = 1), and pain prompting a request for removal (n = 1). The remaining reasons were isolated skin infection (n = 1) and multiple drain dislodgement (n = 1). Common contributing factors included persistent discomfort, recurrent cellulitis, mechanical complications such as clogging or dislodgement, and diminished quality of life, prompting the shift to internal drainage (Table 1).

**Figure 1:**
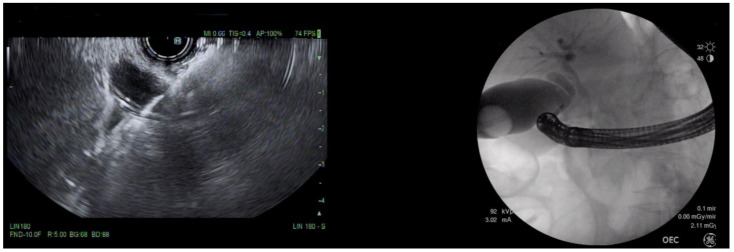
EUS-GBD with PC drain visible on imaging.

**Figure 2:**
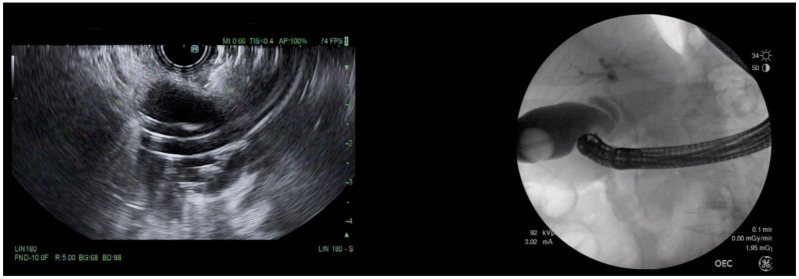
Distal flange deployed.

**Figure 3:**
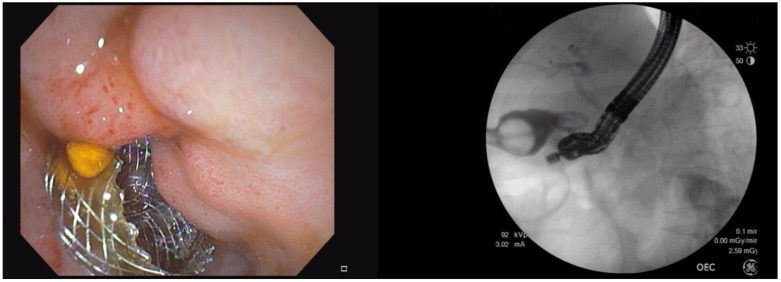
Proximal flange with gallstones emerging.

The EUS-GBD procedure was performed under deep sedation with fluoroscopic and endoscopic ultrasound guidance, utilizing a Gastrovideoscope Ultrasound Curved-linear Transducer (GF-UCT) 180 linear Olympus America scope. After administering prophylactic antibiotics, diluted contrast was injected through the existing PC tube to opacify and distend the gallbladder and confirm patency. Under EUS visualization, the PC drain can be seen anchored in the gallbladder (Figure 1). The gallbladder was punctured trans-duodenally (n = 5) or trans-gastrically (n = 2) using a 10 to 15 mm lumen-apposing metal stent (LAMS), Hot Axios stent by Boston Scientific, which was deployed under both endoscopic and sonographic guidance to ensure proper positioning. A distal flange was first deployed in order to bring the gallbladder closer to the duodenal or gastric wall (Figure 2). Next, a proximal flange was deployed in order to anchor the stent in the gastrointestinal tract, creating a secure connection to allow for bile and stone passage (Figure 3). Stent selection was tailored to individual anatomy, with 15 mm lumen-apposing metal stents used in five cases and 10 mm stents in two cases, all placed under combined endoscopic, sonographic and fluoroscopic guidance. A 7 French x 3 cm double pigtail stent was placed through the Axios stent in 4 patients. The PC tube was clamped and removed a few days later after confirming adequate internal drainage, eliminating the need for external catheter care. Technical success was achieved in all cases (100%), with no immediate procedural complications such as perforation or stent migration. One patient had minor bleeding during the procedure that self-resolved. Patients were monitored post-procedure for adverse events, including stent occlusion, cholangitis, bleeding or perforation, none of which were observed during the follow-up period. Follow-up over a median of 90 days demonstrated sustained benefits of EUS-GBD, with no cases of recurrent cholecystitis or stent dysfunction. While most patients underwent endoscopic evaluation at 1 and 4 weeks, extended clinical follow-ups (up to 12–16 weeks) were performed in all cases with no delayed complications observed during these visits. Procedures were completed within 5–15 min (median 10 min).

A critical component of this study was the evaluation of patient satisfaction before and after conversion from PC to EUS-GBD using a standardized 5-point Likert scale (1 = very dissatisfied, 2 = dissatisfied, 3 = neutral, 4 = satisfied, 5 = very satisfied). The Likert scores were collected by 1 research coordinator at the time of the first clinic visit prior to the EUS-GBD and then subsequently on post-follow-ups mentioned above. The Likert scale was chosen for its ability to transform subjective patient experiences into quantifiable data, allowing for direct comparison between drainage methods. All seven patients in our cohort initially rated their experience with the PC drain as a score of 1 on the 5-point Likert scale, highlighting the profound physical and psychological burden associated with external drainage. The Likert scale was carefully selected for this study due to its well-validated ability to quantify subjective patient experiences and standardize comparisons between treatment modalities. This psychometric tool is particularly valuable in assessing quality-of-life metrics as it captures not only clinical outcomes but also patient perceptions of comfort, convenience, and overall well-being, factors that are crucial when evaluating long-term biliary drainage strategies in high-risk populations. The scale’s structured format allowed capturing patient-centered outcomes that clinical metrics might miss.

## 3. Results

The conversion procedure from PC to EUS-GBD was technically successful in all cases, with the internal stent effectively creating a durable connection between the gallbladder and duodenum or stomach. The cohort comprised high-risk patients deemed ineligible for surgery with significant comorbidities, including advanced malignancies (n = 5), decompensated cirrhosis (n = 1), and severe cardiopulmonary disease (n = 1), all of whom initially underwent PC drain placement as a temporizing measure. EUS-GBD was pursued after failed percutaneous cholecystostomy (PC), with indications including recurrent drain-related complications (pain, infection, clogging, dislodgement) and intolerance to external drainage (cellulitis, mobility impairment, reduced quality of life).

Following stent placement, the external PC drain was removed, and the majority of patients (six out of seven, 85.7%) experienced spontaneous closure of their percutaneous tract within 48–72 h. Two patients required almost a week for complete site healing. All procedures achieved technical success (100%) despite the high-risk patient cohort, utilizing both transduodenal (n = 5) and transgastric (n = 2) approaches. Stent selection was anatomically guided, with 15 mm lumen-apposing metal stents deployed in five cases and 10 mm stents in two cases, all under dual endoscopic-sonographic guidance. The safety profile of EUS-GBD in this study was excellent, with only one minor complication observed among the seven procedures (14.3% complication rate). This single case involved self-limited gastrointestinal bleeding at the Axios stent site that resolved spontaneously without requiring any endoscopic or pharmacological intervention. Importantly, no other adverse events, including stent migration, perforation, cholangitis, or severe bleeding, were reported during the study period.

Overall, all seven patients expressed preference for EUS-GBD over their previous PC drainage, with multiple factors contributing to their improved satisfaction. Patient satisfaction scores improved following EUS-GBD, with five patients rating their experience as a 5 (“very satisfied”) and the remaining two assigning a score of 4 (“satisfied”). Patients reported higher satisfaction post-EUS-GBD (mean score 4.7) compared to PC drainage (mean score 1), with improvements noted in comfort, convenience, and quality of life. The two patients who scored 4 instead of 5 cited minor residual abdominal discomfort resolving in two days, reporting an improvement compared to their prior experience with external drains. During a median follow-up of 90 days, EUS-GBD maintained its therapeutic benefits without recurrence of cholecystitis or stent dysfunction. Initial evaluations occurred at 1 and 4 weeks, with all cases monitored for 12 to 16 weeks. Long-term outcomes revealed no cases of recurrent cholecystitis or stent dysfunction identified during the follow-up period.

## 4. Discussion

AC is one of the more common gastrointestinal diseases requiring surgical intervention seen by physicians in a hospital setting. While LC remains the intervention of choice, other options such as PC drain placement and EUS-GBD have been readily available. Some of the reasons that these latter options are indicated include failure of medical treatment, high-risk surgical candidates, severe sepsis, suspected necrosis or perforation of the gallbladder, patient refusal, advanced age, and more [9]. PC is typically performed by interventional radiologists with the use of ultrasonography or fluoroscopy. It involves puncturing the gallbladder followed by placing a drain catheter to gravity in order to assist with gallbladder decompression [10].

There are two types of methods to conduct a PC. The first one is the transhepatic approach, which involves accessing the gallbladder by inserting a catheter through the liver. The advantage of using this method is that it provides greater stability for the catheter by anchoring it to the liver parenchyma, which reduces bile leakage and faster maturation of the tract [10]. On the other hand, the transperitoneal approach involves inserting a catheter directly into the gallbladder through the abdominal wall. This method may be preferred in patients who have liver disease or coagulopathy [10]. According to a study by Hatzidakis et al., there is no significant difference in complication rates between these two methods [11]. There are no absolute contraindications to proceeding forward with a PC; however, there are a few relative contraindications. Coagulopathy is the most common contraindication, with a goal of >50,000 platelets and INR < 1.5 prior to proceeding with a procedure. Additionally, allergies to iodine may preclude individuals from moving forward with fluoroscopic procedures such as a PC. Anatomical limitations such as ascites or tightly packed gallbladders with stones can make catheter placement difficult, although complication rates are not significantly different in those with ascites vs. no ascites [12].

Some of the more common complications seen in PC procedures include catheter dislodgement, bile leak, and catheter blockage, while more major complications such as sepsis, hemorrhage and bowel injuries are less frequently seen [13]. Tube dislodgement is the most common complication seen during this procedure. Due to the frequent dislodgement of the catheter drain (7–7.6%) and the prolonged duration that the catheter stays in (3–6 weeks), this procedure can potentially lead to patient dissatisfaction [13,14]. While biliary infection and sepsis might be uncommon, the introduction of an instrument to the skin barrier exponentially increases the incidence of sepsis, especially in the elderly, immunocompromised, and patients with multiple comorbidities [15].

While acute cholecystitis is the most common type of pathology seen in hospital settings, acalculous cholecystitis (ACC) makes up only about 5–10% of all cases seen. This is characteristically described as inflammation of the gallbladder without the presence of any gallstones [15]. This condition is typically seen in critically ill patients in the intensive care unit recovering from major surgeries, heart attacks, strokes, sepsis, burns, and more [16]. While the original Tokyo guidelines in 2013 considered gallbladder drainage as mandatory for those patients with severe ACC or moderate severity in those who failed initial therapy, the Tokyo guidelines in 2018 proposed that these severely ill patients may potentially be treated with LC [17]. A systematic review conducted by Ambe et al. looked at 337,500 patients and concluded that there was no reported benefit for the use of percutaneous transhepatic gallbladder drainage (PTGBD) over LC [18]. The use of PTGBD compared to its surgical counterpart was associated with longer hospital stays, increased mortality and readmission for gallstone-related diseases [18]. This begs the question of whether PC should really be the gold standard intervention for critically ill patients. To answer this question, the use of PTGBD vs. LC in critically ill patients was further studied in a randomized controlled trial called the CHOCOLATE trial in 2018. The study found that LC was superior to PTGBD in those patients who had ACC [19]. In addition, patients who underwent LC had fewer major complications when compared to those who received PTGBD (65% vs. 12%) [19]. In addition, 66% of the PTGBD group required reintervention, while only 12% of the LC group did. Recurrent biliary disease occurred much more often in the PTGBD group (53% vs. 5%), and the median length of stay in the hospital was longer (9 vs. 5 days) [19]. The trial ultimately concluded that LC use in high-risk patients with ACC was preferred compared to PTGBD. This study suggested that even in critically ill patients, LC was preferred if patients were able to tolerate surgical intervention.

In comparing PC to EUS-GBD, both retrospective comparative studies and meta-analysis studies show that EUS-GBD is generally associated with better overall outcomes. Some of these examples include lower readmission rates, reintervention rates, recurrence of acute cholecystitis and significantly lower adverse effects at the 1-year mark [20]. Specifically, a systematic review and meta-analysis of 1155 patients conducted by Hemerly et al. showed that EUS-GBD had a better safety profile, less recurrent acute cholecystitis, and fewer hospital readmission rates when compared to patients who underwent PC [21]. Additionally, Teoh et al. compared 59 patients who received a PC and 59 patients who received EUS-GBD. This study found that 44 patients (74.6%) in the PC group suffered from adverse effects, while only 19 patients (32.2%) in the EUS-GBD group suffered from adverse effects [22]. The rapid resolution of the external drain site further reinforces the advantages of internal drainage, as it eliminates ongoing wound care requirements and reduces infection risks associated with long-term external catheter use. External PC drains are frequently associated with considerable physical discomfort, restricted mobility, recurrent infections at the insertion site, and the need for frequent dressing changes, all of which substantially diminish quality of life. In contrast, EUS-GBD provided a cosmetically preferable, internalized solution that required no external maintenance. Patients particularly appreciated the absence of an external catheter, which allowed them to resume normal daily activities, bathe without restrictions, and avoid the social embarrassment or inconvenience of a visible medical device.

EUS-GBD is a fairly novel intervention that is safe and minimally invasive and could be used as an alternative to PC. This procedure is performed by an interventional gastroenterologist under endoscopic, EUS and fluoroscopic guidance [23]. One of the major concerns for endoscopists is maintaining that fistulous tract without the risk of biliary leak. The incidence of biliary leak after the use of LAMS is extremely rare due to the opposing force generated by this specific stent [24]. At this time, there is no data to suggest how long a metal stent needs to remain following the initial EUS-GBD. However, a multicenter retrospective study conducted by James et al. suggests that metal stents are typically replaced by a plastic stent within 2–4 weeks to allow for tract maturation and continuous drainage [25]. However, for certain patients who do not elect for a follow-up endoscopic procedure, a metal stent can remain in the lumen to allow for passage of bile and stones [26]. Ishii et al. demonstrated 100% clinical success in 35 patients who underwent EUS-GBD [27]. In a systematic review and meta-analysis conducted by Boregowda et al., they highlighted that patients who underwent EUS-GBD had better technical success, fewer adverse effects, and lower reintervention rates [28].

The future direction on the use of EUS-GBD relies on many ongoing factors. Establishing standard protocols on how to conduct the procedure and picking the right patient population will allow clinicians to ensure consistent, high-quality outcomes. In addition, expanding the current indications of EUS-GBD may include definitive therapy, a bridge to surgery, and stone extraction through LAMS placement. Ongoing advancements in stent designs, such as LAMS, will allow for improved safety, decreased adverse effects, and standardization of stent selection. The need for more prospective studies on the safety and long-term efficacy of these stents is needed in order to establish this standardization. This includes the length that stents need to stay in place and whether they need to be removed or replaced by a different stent. Our study provides evidence of improved patient satisfaction with EUS-guided gallbladder drainage compared with alternative treatment approaches. Larger, prospective studies are needed to further evaluate patient satisfaction as a key clinical outcome following EUS-guided gallbladder drainage.

## 5. Limitations

This study has several limitations. First, it was designed as a pilot study with a limited sample size. Second, the retrospective case series design may introduce selection and observational bias. Additionally, all procedures were performed by a single operator, which may limit the generalizability and reproducibility of the findings across different practice settings. Some additional information regarding acute cholecystitis severity, gallstone status and anticoagulation status was not available to report. Future prospective randomized controlled trials with two study arms are needed to further validate these results and better assess the efficacy and tolerability of the procedure.

## 6. Conclusions

In summary, this multi-center study provides compelling evidence supporting the use of endoscopic ultrasound-guided gallbladder drainage (EUS-GBD) over percutaneous cholecystostomy (PC) in terms of patient satisfaction among individuals who are not candidates for surgical intervention. The unanimous dissatisfaction with PC and the marked satisfaction with EUS-GBD, as reflected by the 5-point Likert scale ratings, highlight a shift in patient experience and preference when transitioning from a traditional, more invasive approach to a minimally invasive, endoscopically guided technique. These findings are particularly noteworthy given the vulnerable nature of the patient population studied, those for whom laparoscopic cholecystectomy is not an option due to comorbidities or other contraindications. The discomfort and adverse effects associated with PC have long been recognized, but alternatives have been limited. EUS-GBD, as demonstrated in this study, not only addresses these limitations but also offers a substantial improvement in quality of life and overall patient well-being.

While the sample size is small, the results are consistent across multiple institutions. Furthermore, the collection of demographic and clinical data provides a foundation for future research to explore long-term outcomes, cost-effectiveness, and potential complications associated with this novel approach. Given the high satisfaction scores and the minimally invasive nature of EUS-GBD, it is reasonable to advocate for its consideration as a non-surgical intervention in patients with acute cholecystitis who are not suitable candidates for laparoscopic cholecystectomy. Continued research with larger cohorts and longer follow-up periods will be essential to further validate these findings and to refine patient selection criteria. Nevertheless, this study marks an important step toward improving the standard of care and patient experience in the management of acute cholecystitis among high-risk populations.

In conclusion, this multicenter retrospective case series demonstrates that conversion from percutaneous cholecystostomy to endoscopic ultrasound-guided gallbladder drainage was technically feasible in seven selected high-risk patients and was associated with favorable short-term patient-reported satisfaction. While these findings suggest that EUS-GBD may represent a valuable alternative to external drainage in carefully selected nonsurgical candidates, the small sample size and retrospective design limit definitive conclusions. Larger prospective studies are needed to further evaluate long-term outcomes, safety, and patient-centered benefits of this approach.

## Figures and Tables

**Table 1:** Summary of Clinical Characteristics, Procedural Details, and Outcomes of Patients Undergoing EUS-Guided Gallbladder Drainage (EUS-GBD) Following Percutaneous Cholecystostomy (PC).

A.	Sex	Comorbidities	Reason for PC	Reason for EUS GB Drainage	Type of Scope	Approach Transduodenal (D), Transgastric (G)	Stent Type	Stent Size	Efficacy of EUS-GBD	PC Postoperative Drain Removal	Post EUS-GBD Complications	Follow-Up (Duration in Weeks)
81	F	Pancreatic cancer with Liver metastasis (Stage IV)	Not deemed a surgical candidate	Skin infection	GF-UCT180 linear Olympus America	D	Axios-Boston scientific	15 mm	Successful	48 h	Pain	1, 4, 12
63	F	HCC (Stage IV)	Not deemed a surgical candidate	Pain and excoriations at the site of the PC drain	GF-UCT180 linear Olympus America	D	Axios-Boston scientific	10 mm	Successful	72 h	None	1, 4, 12
89	M	Pancreatic cancer with Liver metastasis (Stage IV	Not deemed a surgical candidate	Pain and drain malfunction	GF-UCT180 linear Olympus America	G	Axios-Boston scientific	15 mm	Successful	48 h	None	1, 4, 12
70	F	Cirrhosis with ascites (MELD 26)	Not deemed a surgical candidate	Pain and site infection	GF-UCT180 linear Olympus America	D	Axios-Boston scientific	15 mm	Successful	7 days	None	1, 4, 16
48	F	Breast Cancer with liver metastasis (Stage IV)	Not deemed a surgical candidate	Pain and patient intolerance	GF-UCT180 linear Olympus America	D	Axios-Boston scientific	15 mm	Successful	48 h	Pain	1, 4, 8, 12
76	M	COPD, CAD, CHF (NYHA III)	Not deemed a surgical candidate	Multiple drain dislodgement	GF-UCT180 linear Olympus America	G	Axios-Boston scientific	15 mm	Successful	7 days	None	1, 4, 16
92	F	Pancreatic cancer with Liver metastasis	Not deemed a surgical candidate	Pain and the patient’s request for removal	GF-UCT180 linear Olympus America	D	Axios-Boston scientific	10 mm	Successful	8 days	None	1, 4, 12

F: Female; M: Male; PC: Percutaneous Cholecystostomy; IV: Intervenous; HCC: Hepatocellular Carcinoma; MELD: Model for End-Stage Liver Disease; COPD: Chronic Obstructive Pulmonary Disease; CAD: Coronary Artery Disease; CHF: Congestive Heart Failure; NYHA: New York Heart Association; GF-UCT: Gastrovideoscope Ultrasound Curved-linear Transducer.

## Data Availability

The original contributions presented in this study are included in the article. Further inquiries can be directed to the corresponding author (Kamran Ayub).

## Abbreviations

The following abbreviations are used in this manuscript:

AC	Acute Cholecystitis
ACC	Acalculous Cholecystitis
ASA	American Society of Anesthesiologists
CAD	Coronary Artery Disease
CHF	Congestive Heart Failure
COPD	Chronic Obstructive Pulmonary Disease
EUS	Endoscopic Ultrasound
EUS-GBD	Endoscopic Ultrasound-Guided Gallbladder Drainage
HIDA	Hepatobiliary Iminodiacetic Acid (scan)
INR	International Normalized Ratio
LAMS	Lumen-Apposing Metal Stent
LC	Laparoscopic Cholecystectomy
NOM	Non-Operative Management
PC	Percutaneous Cholecystostomy
PTGBD	Percutaneous Transhepatic Gallbladder Drainage
